# Experiments and Observations on Mesmerism

**Published:** 1829-10

**Authors:** Richard Chenevix


### MESMERISM.

*Experiments and Observations on Mesmerism.* By RICHARD CHENEVIX, Esq. F.R. and E.S., M.R.I.A., &c. (5th and last Article.)

THE experiments which I now have to relate were made in presence of Dr. ELLIOTSON. The following notes were drawn up by him each day, and I transcribe them with a few verbal alterations, and remarks, to elucidate some points.

Dr. Elliotson says, "On May 14th, 1829, I was introduced to Mr. Chenevix, at the Royal Society, by Dr. Hodgkin, and invited to witness the phenomena of mesmerism, the next morning, at Mr. Chenevix's lodgings in Old Burlington street. On the following morning I found two females,* said to be sisters, sitting in two chairs side by side; the one apparently about eighteen years of age, of a pretty full habit, but a colourless and heavy countenance; the other apparently about twelve years old, with a fine skin, florid complexion, and animated expression.

"Mr. C. asked the elder whether she had any pain? She replied, 'Yes, in her forehead.' He passed his hands before her forehead two or three times, and I believe placed one of his fingers on it; when she said the pain had moved to the top of her head. The same operation was repeated on the top of her head; when she said the pain was gone. Mr. C. then began to draw down his hands before her, in the usual way of magnetisers, and frequently in contact with her face and clothes, and likewise placed his hands motionless on the front of the body and the loins, sometimes at the same moment; and in three or four minutes she looked sleepy, when he gently pressed down her eyelids, and inclined her head to one side, as if to place it for support against a piece of wood at the back of her chair. She remained for a considerable time, as far as I could judge by very carefully watching her features and her breathing, in a sound sleep; and was at last awakened by Mr. C., as any person might be awakened by another.

"As soon as this girl appeared fairly asleep, Mr. C. began the process with the younger sister; but, though she soon appeared sleepy, she was as long again in assuming the same appearance of sleep as the first; and, as I had placed my chair at the side of Mr. C. close to her, and consequently could not make very minute observations on the elder, I resolved to satisfy myself, as fully as I could,

* Mary Ann and Sarah H., mentioned in the last article.

that the little one played no tricks; and, if she had not shown more marks of sleep than she did for the first few minutes that her head reclined to one side, in the attitude of repose, I should have remained doubtful of the power of the manipulations. But at length, perhaps in about seven or eight minutes from the commencement,* I was as satisfied of her being in a sound sleep as a bystander could be. She did not waken until roused by Mr. C., as her sister had been roused. Her eyes and cheeks were then red, and her eyes heavy; exactly as is observed in persons really awakening from sleep. While they were both apparently asleep, Drs. Wright and Wilmot entered the room.

Mr. Chenevix was requested to attempt to throw the younger one into sleep again. He did attempt it during about four minutes, and she fell into the same appearance as in the beginning of the former process; and, on being questioned, she very ingenuously answered that she had not been asleep this second time.

"On the second morning I took Mr. C. to ——, for the purpose of seeing the process tried upon persons who had never heard of mesmerism, who were ignorant of what was to be done, and had never communicated with Mr. C. Dr. —— met us there, and was requested to send for any patient he thought proper. Mary P., about eighteen years old, was introduced. She was subject to hysteria, and had suffered a paroxysm that morning, and was asleep at the time she was sent for. She appeared a little flurried, and her pulse was 150. Mr. C. had scarcely touched her when an hysterical paroxysm came on, and lasted for an hour; during the whole of which the process of mesmerism was continued by Mr. C., who frequently attempted, but in vain, to calm and rouse her. She was perfectly insensible during the whole hour, but the fit was less violent (said Dr. ——) than ordinary. A second fit, too, he informed us, rarely happened in one day. I could discern in this nothing but an hysterical paroxysm induced by agitation of mind. Mr. C. frequently attempted to remove the rigidity of her arms, and to relax the fingers by mesmerism, without the least effect.

"I now sent for a little girl labouring under chorea, but who was nearly cured by subcarbonate of iron. The same manipulations were tried for ten minutes, but not the least effect was produced.

* She was, in truth, asleep, as usual, in one minute; though, to a person who saw these experiments for the first time, she might not appear so.

"I sent for two other females, about eighteen or twenty years old, on both of whom Mr. C., judging from the tremulous motion of their eyelids, hoped for success; but the manipulations of mesmerism, continued for ten minutes, had no effect. One of them, indeed, questioned by Mr. C., said she felt her head light. He then, without speaking, moved his fingers before her forehead: she said it felt heavy. These sensations alternated four times, till, after a last manipulation, she declared her head to be quite well.

"Dr. —— then brought in Emma W., about twenty-five in appearance, and subject to epilepsy; her age was, in fact, but eighteen. In ten minutes her eyes closed, and her head suddenly dropped forwards; but I did not consider her to be asleep until ten minutes more had elapsed. She then really seemed in sound and tranquil sleep. Her hands, when raised, dropped immediately; her eyes were completely closed. The whole frame remained motionless. I placed my face close to hers, for the purpose of seeing if her eyes were quite shut, and she did not move a feature; neither did the friction of her eyelashes cause contraction in any muscle. On being woke by Mr. C., the redness of her eyes and cheeks, and the heaviness of her look, were completely those of a person wakening out of sleep. Before she appeared to waken, Mr. C. asked her some questions, which she answered; and, while the conversation proceeded, she became gradually, as it appeared, quite awake. Mr. C. declared her to have been asleep, and her first answers, to have been given by her in magnetic sleep. He augured from this that in time lucidity might be manifested.

"May 17th, carried Mr. C. again to ——, where he proceeded to mesmerise the hysterical girl, Mary P. He said that, to his mind, it was clear that the paroxysm of yesterday was the effect of mesmerism; and he was inclined to think that, should another be brought on today, it would be slighter. He looked upon this patient as the most susceptible he had yet met with in London. She was much calmer to-day on coming into the room than yesterday: her pulse was 130. She presently closed her eyes, and her head went from side to side, as that of a person asleep; and I felt satisfied that she was so. In a few minutes convulsive motions began, and continued near an hour. Dr. —— said that her convulsions were generally preceded by a short drowsiness and insensibility. At length the convulsions became very violent, and Mr. C. again and again attempted, in vain, to tranquillize her, to calm her arms, legs, &c.; and we thought it prudent to stop the process. She was carried to her bed, strapped down upon it, and in about five minutes she returned to herself, and became calm.*

"Mr. C. then mesmerised Emma W. In about the same time as yesterday she looked asleep; her hands, when raised, dropped as before; and Mr. C. signified that he thought her to be asleep. She remained so for ten minutes, Mr. C. proceeding as he expected her to answer him in that state. When he addressed her, however, she spoke as if awake, and assured us that she had not been asleep, either to-day or yesterday; contrary to Mr. C.'s expectation. She said that both times she felt drowsy. He then mesmerised her arm, with the intention, as he said, of paralysing it. She said it pained her. After a few transverse passes, she said the pain was gone. The same effects were produced, and by the same means, on her head. He then placed a piece of paper (as related in a former article) on one of her arms, and desired her to raise them both. She felt some difficulty in raising that on which he had put the paper. When she had walked about half-way across the room to go away, he darted his hands violently towards her feet. No effect ensued. Mr. C. said that he had often arrested the progress of his mesmeric patients, and rendered them motionless, by a similar process.

"May 18th.—The chorea girl, who had felt her head successively light and heavy on a former day, had commenced menstruating this morning, though she had not done so for several months. Three more female patients were submitted to the process, but with no apparent result.

"A fourth patient was now seated in the chair. She exhibited no apprehension of any kind, but was talking very cheerfully to me. Mr. C., without saying one word to her, began his manipulations, at the distance of half a foot, but did not touch her. In about one minute she said, in a plaintive voice, "Sir, don't do that;" and seemed in great distress. She afterwards told us that Mr. C. drew weakness

* My opinion upon this patient is, that both these paroxysms were induced by mesmerism. I have generally seen, too, that the intensity of such attacks diminishes each time, while the patient is undergoing the process. A girl, aged ten, had eleven fits the first day I mesmerised her, all, from first to last, decreasing in violence and duration. The second day she had three, and never after any. Had the patient, whose case is here recited by Dr. Elliotson, been mine, I would not have permitted any one to touch her, but would have continued the operation that day, and afterwards. Persons unacquainted with the extraordinary effects of mesmerism may, however, well be alarmed at them; and the prudence of these gentlemen was highly commendable.

into her, and made her feel faint. She complained of pain in the abdomen. Mr. C. moved his hands transversely before it, and she said the pain was gone. (She had felt a slight pain there before we saw her.) She then complained of great uneasiness in her chest; and after some transverse movements, made by Mr. C. with the intention of removing it, she declared it was gone. The pain in the abdomen returned and ceased, as before, by the manipulations of Mr. C. Mr. C. then darted his open hand towards one arm, without touching it, and told her to raise both arms. She scarcely could move that which he had thus mesmerised. He then made some transverse passes before it: she at once moved it, and declared the stiffness and uneasiness to be gone. The same was repeated with the other arm, and with the same effect. He told her to lift her feet: she did so with perfect ease. He then darted his hand toward one leg, and she stared with astonishment at finding that she could not stir it without the greatest difficulty. He then made some transverse passes, when she instantly raised it, and said there was neither pain nor stiffness in it. He then closed her eyes, and put a very small piece of paper, weighing perhaps one grain, on her foot, in such manner that it was utterly impossible she could perceive it: she could scarcely move that foot. The paper was removed in the same manner, and without her knowing it: she could instantly raise her foot. She now complained of pain about the heart: Mr. C. demesmerised her, and she said it was gone. In all these experiments, Mr. C. had most clearly announced to me, in French, what his intentions were; and the effects coincided so accurately with those intentions that I confess I was astonished. Deception was impossible. Mr. C. looked round at me, and asked, in French, if I was satisfied. I really felt ashamed to say no, and yet I could scarcely credit my senses enough to say yes. I remained silent. He then asked me, still in a language unintelligible to the patient, 'Shall I bring back a pain or disable a limb for you once more?' I, of course, requested that he would do so. He complied instantly, giving her a pain in the chest once, and disabling her several times from moving her limbs, and removing those effects at pleasure, according to the intentions which he announced to me; the whole taking place exactly as it had done in every former trial on this woman. As, however, she began to feel faint and uncomfortable, Mr. C. judged it prudent to desist; assuring me that such experiments as these should never be repeated, but with moderation, and only by experienced mesmerisers.

"On questioning this woman a few days after Mr. C. had produced such decided effects upon her, respecting what had occurred, she declared that he had disabled first one limb, then another, and restored their use, exactly as appeared to be the case; that she never had felt any thing like it in her life before; that, though she had not slept during the operation, she had felt very drowsy; that she had not been at all afraid; but, said she, 'I hope never to see that doctor again, as I am sure he has something to do with the devil.'

"On May 20, I witnessed some experiments on another patient, at Mr. C.'s lodging, in company with Dr. Wright; but the results were not satisfactory."

Here ends Dr. Elliotson's narrative.

The persons who have given up the most time and attention to such mesmeric experiments as I could make in London, were Drs. Whymper and Elliotson, with Mr. Smith; and they, too, have witnessed the most extraordinary phenomena. Other persons saw but one or two trials, and with five to one against success, that number is small. I will, however, conclude the subject by summing up and discussing the evidence of all; for, like every other evidence, this must be appreciated not piecemeal, but in toto.

Confining myself to the experiments made in London; admitting five failures to one success; bearing in mind that one positive experiment can do more to support a fact than ten negative experiments can do to subvert it; that "plus valet unus affirmans quam mille negantes;" I proceed to say that I can quote, as witnesses to the mere fact of sleep, the Marquess of Lansdowne, Dr. Babington, jun. Drs. Elliotson, Hargood, Holland, Milligan, Prout, Whymper, Wilmot, Wright, Messrs. Brodie, Smith, Bagnold; as witnesses of other effects, Drs. Elliotson, Milligan, Whymper, Messrs. Earle and Riadore Smith: fifteen most credible men, or there are none in the world. These credible and highly enlightened men, then, admit the facts which they saw; but, as is very natural on so novel a subject, explain them by various theories. Dr. Prout, who is not prepared to admit that my action upon Sarah H. was the cause of her sleep, or to believe in mesmerism, is yet at a loss to know to what other agent but me, he can ascribe that sleep. Mr. Brodie explains it as he does giddiness by a rotatory motion, or sleep produced by rocking a child. Lord Lansdowne and Dr. Holland attribute it decidedly to me, but deny the existence of any new agency. Dr. Hargood attributes all to the imagination of the patient, influenced by me. Dr. Milligan, struck by the promptness of the effect, admits that he cannot explain it by any of the usual modes which produce sleep, but inclines to admit a peculiar influence exercised by me. Mr. Evans Riadore was not very remote from a similar opinion. Mr. Earle, giving ample and able testimony of facts, is more a believer in the operation of the patient's mind than of my mesmerism. Drs. Whymper and Elliotson, and Mr. Smith, attempt no explanation; but the facts which they saw need no comment; for it is absolutely impossible to explain them by any known agency. It will be sufficient, then, to prove that these effects are inexplicable by any of the hypotheses advanced by the persons above quoted, in order to prove that what they saw is also inexplicable by their hypotheses. Imagination shall be first discussed.

Imagination cannot act in conformity with the expectations of the operator, unless it has been previously told what is expected from it. Now, even admitting the minds of Garrand and Gould, in the Coldstream Guards hospital; of the woman at St. Bartholomew's hospital; of the woman whose case has been just stated by Dr. Elliotson, to have been prepared for *some* result, still it was impossible for these patients to guess *what* my intention was in each experiment. Yet their sensations, by the fair avowal of Dr. Whymper and Mr. Smith, of Mr. Earle, and of Dr. Elliotson, most accurately corresponded with the intentions which I had previously announced each time. All these witnesses, too, strictly watchful to prevent deception or collusion, confess that nothing of the kind could be: for, till the minute of trial, I had never seen any of these patients; had not, for twenty years, been in any of the hospitals where I operated; and could not know what persons would be selected for trial, since the physicians themselves did not know whom they would choose. Imagination, then, cannot be admitted as the agent by which these effects were produced. Should a patient as often divine the operator's will as occurred in these experiments, the wonder would be as great as if the coincidence resulted from a new agency; for his mind must, indeed, be endowed with supernatural sagacity.

Mr. Brodie saw but one experiment and one result, sleep; the least interesting and the least convincing of any. But let him admit the evidence of Messrs. Earle and Smith, of Drs. Whymper and Elliotson, (and surely we must sometimes credit each other's testimony;) let him connect their facts with his facts, as proceeding from one cause. How will he explain this truth: that, at my will, whichever limb I pleased, of a woman whom I had never beheld till that moment, was deprived of motion and restored to motion instantly, without my giving that woman any intimation of what my will was? That I gave her pains, and took pains away from her, as I pleased, she being ignorant of my intention? That Garrand was almost nailed to his chair by the same means? That Gould was wakened from profound sleep by transverse passes made behind his back, unknown to him? The rocking of a cradle could not do all this. Mr. Brodie diffidently says, "With such information as I at this moment possess, &c." Most heartily do I regret that his occupations did not permit him to follow up the subject more particularly; for no testimony can be of higher value than his. Had he seen the experiments here alluded to, his rationale of the facts would have been very different.

Upon the whole, sufficient time could hardly be allotted by any of the persons here named to these researches; and they were attempted under great disadvantages from that circumstance.* Things which are so directly opposite to current opinions should be witnessed more than once before they are judged. Could these phenomena be infallibly produced at will, and before an unlimited number of witnesses, the question would be decided at once. Sentence might long since have been pronounced upon a public theatre. But they are too delicate for common exhibition.

To combat all evidence on a subject which seems so marvellous, to destroy the value of every testimony, they who think that no man can see but themselves, adduce the many errors and impostures which have gained evidence in the world for a time, and then have been exploded. They appeal to the fables of antiquity, to the superstitions of Mahomet, to the savages of Africa. But can any man of good faith compare the principles and conduct of mesmerisers with those? The ancients knew not what the diffusion of knowledge was, and science was a secret of priestcraft. Mahomet founded an empire and a sect, and these two great prizes of his ambition rewarded the lies and devastation which his military apostles spread through the world. Savages, at all times, were the easy dupes of superstitious credulity. But the partizans of mesmerism,

* It is impossible to give an elementary course of mesmerism in these articles; but the "Instructions Pratiques," by M. Deleuze, in one small vol. 8vo., more than supply every deficiency.

stimulated by no interest but truth, appeal not to an ignorant multitude, easy to deceive or to fanaticise; but to men of science and genius, scrutinizers of nature, ponderers upon her works. Would the author of the "Mecanique Celeste," which not fifty men living can comprehend, condescend to lay his opinions bare to umpires so much his inferiors as they would be, whom mesmerism could this day deceive? Would all the learned disciples of this art in Europe give up their honest fame for the reputation of impostors? Some antagonists avail themselves of the discredit into which Mesmer fell by his own doctrines. But this is a fallacious mode of reasoning; for, if his doctrines are true, Mesmer is absolved from this imputation. Mesmer was, indeed, declared to be the greatest mountebank of the last century; but by whom? by the greatest mountebank of modern history, Bonaparte. That Mesmer was disinterested, a profound philosopher, an original discoverer, cannot, indeed, be supported by the history of his life: his rapacity was excessive; his injustice, in not sufficiently acknowledging the prior claims of Maxwell, Greatreakes, Gassner, &c. was extreme; his own title to the doctrines which he taught was small. But those doctrines are independent of his talent or his veracity: they stand upon their own merits; they have been proved by thousands; and, if his name has here been affixed to them, it is the hope that the appellation of animal magnetism, now deemed to convey an erroneous notion of this agent, may be discarded.

How strangely must they estimate nature, how highly must they value themselves, who deny the possibility of any cause, of any effect, merely because it is incomprehensible? For, in fact, what do men comprehend? Of what do they know the causes? When Newton said that gravitation held the world together, did he assign the reason why the heavenly bodies do not fly off from each other into infinite space? He did but teach a word; and that word has gained admittance, as it were, surreptitiously amid causes, even in the minds of the most enlightened, insomuch that to doubt it now were a proof of ignorance and folly.

Let an untutored Indian hear, for the first time, that the moon, which rolls above his head, is suspended there by the power of gravitation; that she obeys the influence of every little speck which his eye can discern in the firmament; of orbs placed beyond them again, but invisible to us, because their light has not yet reached our globe; that the earth cannot be shaken, and the shock not communicated through the whole system of the universe; that every pebble under his feet as virtually rules the motions of Saturn as the sun can do. Let him then be told that one sentient being, placed in the vicinity of another sentient being, can, by a certain action of his nervous system, produce the daily phenomenon, sleep, and the rarer one, somnambulism; and which of these lessons would he be the most prompt to credit? Certainty not that which inculcates an impalpable action and reaction between infinite masses, separated by infinite distances. The pride of learning, the arrogance of erudition, deem it ignoble to believe what they cannot explain; while simple instinct, struck with awe by every thing, is equally open to credit what it cannot as what it can comprehend, and admits no scholastic degrees of marvellousness.

Whatever explanation be given of mesmeric phenomena by those who have seen them once, or by those who have seen them repeatedly, still the facts remain the same; and these are the truly precious parts of every science. If medical men assert that the alleged cures of mesmerism are performed by the mind, and that this is the peculiar province of imaginative therapeutics, do they not culpably neglect the most powerful agent of mental medicine, if they do not practise mesmerism? If imagination can cure, and if this be its most energetic exciter, then excite it thus; cure by imagination, and the sick will bless you. If the cause be analogous to a rotatory or a rocking motion, then whirl or rock your patients into sleep and health. If it be a new agency, find it out, and prove it by experiment. Many of the persons named in these articles have promised to put it to this test. I here summon them to fulfil that promise, and to fulfil it speedily. The interest of science demands it, whether it be to establish a truth, to subvert an error, or to detect an imposture. All my hope is that ere long the public may hear from them and others, that "mesmerism is true"—or false.

